# Current Utility of Sequential Organ Failure Assessment Score: A Literature Review and Future Directions

**DOI:** 10.2174/1874306402115010001

**Published:** 2021-04-13

**Authors:** Rahul Kashyap, Khalid M. Sherani, Taru Dutt, Karthik Gnanapandithan, Malvika Sagar, Saraschandra Vallabhajosyula, Abhay P. Vakil, Salim Surani

**Affiliations:** 1 Department of Anesthesiology and Perioperative Medicine, Mayo Clinic, Rochester, MN 55905, USA; 2 Department of Internal Medicine, Jamaica Hospital Medical Center, Jamaica, NY 11418, USA; 3 Corpus Christi Medical Center, Corpus Christi, TX 78411, USA; 4 Department of Neurology, Mayo Clinic College of Medicine, Mayo Clinic, Rochester MN, USA and Hennepin County Medical Center, Minneapolis, MN 55905, USA; 5 Department of Internal Medicine, Yale-New Haven Hospital and Yale University School of Medicine, New Haven, CT 06510, USA; 6 Department of Pediatrics, McLane Children’s Hospital, Baylor Scott and White Health, Temple, TX 76502, USA; 7 Critical Care Medicine, Mayo Clinic College of Medicine, Mayo Clinic, Rochester, MN 55905, USA; 8 Texas A&M University System Health Science Center, Bryan, TX 77807, USA

**Keywords:** SOFA, Critical care medicine, Prognostication, Mortality prediction, Intensive care unit, Chronic liver failure

## Abstract

The Sequential Organ Failure Assessment (SOFA) score is commonly used in the Intensive Care Unit (ICU) to evaluate, prognosticate and assess patients. Since its validation, the SOFA score has served in various settings, including medical, trauma, surgical, cardiac, and neurological ICUs. It has been a strong mortality predictor and literature over the years has documented the ability of the SOFA score to accurately distinguish survivors from non-survivors on admission. Over the years, multiple variations have been proposed to the SOFA score, which have led to the evolution of alternate validated scoring models replacing one or more components of the SOFA scoring system. Various SOFA based models have been used to evaluate specific clinical populations, such as patients with cardiac dysfunction, hepatic failure, renal failure, different races and public health illnesses, *etc*. This study is aimed to conduct a review of modifications in SOFA score in the past several years. We review the literature evaluating various modifications to the SOFA score such as modified SOFA, Modified SOFA, modified Cardiovascular SOFA, Extra-renal SOFA, Chronic Liver Failure SOFA, Mexican SOFA, quick SOFA, Lactic acid quick SOFA (LqSOFA), SOFA in hematological malignancies, SOFA with Richmond Agitation-Sedation scale and Pediatric SOFA. Various organ systems, their relevant scoring and the proposed modifications in each of these systems are presented in detail. There is a need to incorporate the most recent literature into the SOFA scoring system to make it more relevant and accurate in this rapidly evolving critical care environment. For future directions, we plan to put together most if not all updates in SOFA score and probably validate it in a large database a single institution and validate it in multisite data base.

## 1 INTRODUCTION

Initially developed to quantify the severity of sickness in sepsis, the Sequential Organ Failure Assessment (SOFA) score is now being used for assessing organ dysfunction in several different Intensive Care (ICU) settings, including medical, surgical, cardiac, neurological, transplant, respiratory care and step down units [1]. Although developed primarily for prognostication, the organ dysfunction, as quantified by the SOFA score inevitably correlates with survival [1]. In addition to being used as an organ dysfunction assessment from underlying disease processes, it is also used for effective resource allocation between different hospital units, to triage patients to an appropriate level of care and as a quality improvement measure [2, 3]. The original SOFA score (**Table S1**) assigns between 0 to 4 points to each of the six individual organ systems, with a total score ranging from 0 to 24. These six organ systems are: respiratory, cardiovascular, neurological, hepatic, renal and coagulation. SOFA score could be calculated every 24 hours of ICU stay. It has been shown to be effective in the prediction of mortality in a variety of patients admitted to the ICU, including acute myocardial infarction [4], post-cardiac arrest syndrome [5] and those requiring extracorporeal cardiopulmonary resuscitation [6]. Several modifications have since been made to the SOFA score (Tables **1** and **2**) in an attempt to simplify it and improve its accuracy. The aim of this study is to conduct a comprehensive review of modifications in SOFA score in the past several years.

### 1.1 Utility of Original SOFA and its Different Derivatives

Studies suggest that the original SOFA score as well as its derivatives have an excellent ability to discriminate between survivors and the non-survivors [3]. The area under the receiver operating curve (AUC) for the SOFA has ranged from 0.75 to 0.90 in various studies [3]. This does not differ from those of other traditional models like acute physiologic and chronic health evaluation II (APACHE II) score and Simplified acute physiology II (SAPS II) score (AUC of 0.80 for APACHE II, 0.83 for SAPS II [7]. The SOFA-based models and derivatives offer an advantage over these other models, as they do not consider chronic comorbidities and reason for admission [3, 8].

The daily-assessed SOFA scores denote improvement or worsening of the involved organ systems and thereby provide information regarding the course of treatment [9, 10]. Change in the SOFA score, i.e. delta SOFA is indicative of worsening organ function and was expected to be highly predictive of poor survival [11]. However, studies have shown that delta SOFA alone has poor discriminating ability between survivors and non-survivors [3]. It has been suggested that delta SOFA may be relatively low in patients with high SOFA score on admission [12, 13]. Several models combining maximum SOFA or delta SOFA over a pre-specified duration with admission SOFA or admission APACHE II and SAPS II scores have been developed [3]. Such combination models have a greater prognostic prediction than either model alone. Additionally, the scoring system cannot be analyzed as a mathematical model. Scoring is a list of the numbers based on validated organ support surrogates. The number itself does not have any mathematical meaning, thus the score should not be seen as linear variable. Here forth we would like to discuss modifications to SOFA score since its inception and point out the knowledge gap.

### 1.2 Modified SOFA [mSOFA] Score

With an aim to simplify the existing SOFA scoring system and devise a score that can be determined directly by the use of electronic records, *Nates et al.* described the mSOFA score in oncology patients [14]. The neurological component is the only component on the original SOFA scoring system that requires a physical examination [15]. Thus this modified score omitted the neurological component. All the other parameters (the laboratory and the vital sign data) can be extracted from an electronic medical record system [14, 16]. Modifications were also made to the respiratory and cardiovascular components (Table **1**). The electronically calculated mSOFA score within 24 hours of admission was found to be equally predictive of mortality in critically ill cancer patients as the original SOFA score. The reported AUCs for mSOFA among surgical and medical patients were 0.78 and 0.72, retrospectively [14]. This mSOFA score also referred to as the non-neurologic SOFA score, has been used in patients with severe neurological injury to determine the degree of organ dysfunction [15].

### 1.3 Modified SOFA [MSOFA] Score

Here the goal was to decrease the number of laboratory values required for determining a SOFA score. In the original SOFA, two organ systems (cardiovascular and central nervous system) are assessed clinically and the remaining four (respiratory, hepatic, renal and hematologic) require laboratory values for their assessment. In certain emergent situations, especially those with mass causalities, it might not be feasible to get all the necessary laboratory values [17]. The hematologic component was omitted, the PaO_2_/FiO_2_ ratio was substituted by the SpO_2_/FiO_2_ ratio and the bilirubin levels were replaced by a clinical assessment to determine the presence of scleral icterus and/or jaundice [18, 19]. The measurement of serum creatinine is the only laboratory parameter included in the MSOFA score. The accuracy of MSOFA score to predict mortality in all critically ill patients was found to be as good as the original SOFA score. Upon comparison, SOFA day-1 and MSOFA have 0.83 and 0.84 AUC, respectively, in predicting mortality [17]. The MSOFA is a good triage tool for resource-limited settings and during periods of mass influx of critically ill patients.

### 1.4 Modified Cardiovascular SOFA [mCV-SOFA] Score

The cardiovascular component of the original SOFA score assigns points based on blood pressure and pattern of vasopressor use [2, 20]. However, the use of several vasopressors and inotropes used in current practice as well as lactic acid levels are not accounted for [2]. The cardiovascular component of the original SOFA score was modified, wherein, hypotension was substituted by the shock index and lactic acid values, as well as the use of vasopressors and inotropes were taken into account (Table **2**). The modified Cardiovascular Sequential organ failure assessment (mCV-SOFA) score improved the overall performance of the SOFA score in predicting patient outcomes. The mCV-SOFA better predicted both ICU mortality (AUC 0.801 *vs*. 0.718) and hospital mortality (AUC 0.783 *vs*. 0.651) when compared to SOFA [2]. Even though these findings were reported in a single-center study, this is the only attempt to incorporate the latest clinical knowledge in modifying the cardiac component in SOFA score.

### 1.5 Extra-renal SOFA Score

This score was specifically developed for critically ill pediatric patients on chronic renal replacement therapy. The renal component of the original SOFA scoring system has been omitted [21]. (Table **1**) There is no data regarding its use in the adult population.

### 1.6 Chronic Liver Failure [CLIF-SOFA] Score

Chronic Liver Failure Sequential organ failure assessment (CLIF-SOFA) score was specifically developed to predict short-term prognosis and mortality in patients developing acute chronic liver failure [22]. Several modifications were made to the original SOFA score. The CLIF-SOFA score has been found to be a better prognosticating tool as compared to several traditional scoring systems in patients developing acute on chronic liver failure. When compared to APCHE-II Child-Pugh Score and MELD Score, CLIF-SOFA had higher performance in predicting 28-day mortality, AUC of 0.795, 0.787, 0.739, 0.710, respectively [22]. This attempt underlines the lone efforts to improve neurological, renal and coagulation components of SOFA with the inclusion of hepatic encephalopathy, Renal Replacement Therapy (RRT) and International Normalized Ratio (INR), respectively.

### 1.7 Mexican SOFA [Mex-SOFA] Score

In an attempt to ameliorate the need to perform arterial blood gases and neurological examination, the Mexican Sequential organ failure assessment (Mex-SOFA) was developed [23]. The original SOFA score requires an arterial blood gas to estimate the PaO2/FiO2 ratio for the evaluation of the pulmonary component of SOFA [18, 19]. It has been well documented that SpO2/FiO2 ratio correlates well with PaO2/FiO2 ratio [18, 19]. Performing neurological examination in intensive care units can also be challenging at times due to the frequent use of sedatives [23]. The PaO2/FiO2 ratio was substituted by the SpO2/FiO2 ratio and the neurological component was omitted. The Mex-SOFA score has been found to be comparable to original SOFA score in predicting mortality in critically ill patients with 0.73 and 0.69 ACUC respectively [23].

### 1.8 Quick SOFA [qSOFA] Score

SOFA scores are being more routinely measured for most patients in the ICU both for clinical and research purposes. However, the intricacy of the method, the lack of all the required information in some patients, and concerns that it may result in late identification of patients at risk question its routine utility in clinical practice. The 2016 SCCM/ESICM task force released the “quick SOFA” (qSOFA) to facilitate easier identification of patients potentially at risk of mortality from sepsis [21]. The qSOFA consists of only three components that are each allocated one point (Table **3**). A qSOFA score of ≥2 points indicates organ dysfunction.

Though it simplifies the original scoring system considerably, it is not without limitations. Several studies [24-27] have shown variable sensitivity of the qSOFA in the early detection of critically ill patients at risk of sepsis related death. A meta-analysis reported a pooled sensitivity and specificity of 46% and 86% in predicting short-term mortality, raising concerns over the usefulness of this simple tool in bedside screening and triaging [28].

### 1.9 qSOFA and Plasma Lactic Acid Levels [LqSOFA] Score

To overcome the limitations of the qSOFA, yet retaining the ease of measurement, it has been shown that combining the three variables in qSOFA along with the plasma lactate concentration significantly improved the predictive ability in identifying at-risk patients. Shetty *et al*. [29] used a lactate threshold of >= 2 mmol/L in patients with confirmed or suspected sepsis. The new score (LqSOFA) was shown to be better than qSOFA (AUC of 0.75 *vs* 0.71) in identifying ED patients at risk of adverse events. The addition of lactate to the qSOFA improved the sensitivity to 72%, comparable to the traditional SOFA score [30]. Another study categorized lactate levels into three categories (<2 mmol/L, 2-4 mmol/L and >4 mmol/L). Hence in the ED or in centers where it is impractical to get all the variables for the full SOFA score, the addition of lactate to the qSOFA may prove as a viable alternative [31].

### 1.10 SOFA in Hematological Malignancies [SOFA-HM] Score

In patients with hematological malignancies, *Greenberg et al.* [32] sought to improve the precision of the SOFA by incorporating recent infection as a variable into the scoring system. Apart from the six traditional systems used by the SOFA, the SOFA-HM score also includes infection. Patients with no documented infection within 90 days of an ICU stay were assigned 0 points, those with an infection within 31 to 90 days were assigned 2 points and infection within 30 days or less was given 4 points. The SOFA-HM score was found to perform better than the SOFA (AUC of 0.73 *vs* 0.68) in predicting mortality in this particular patient cohort. The implementation is a little challenging given that it cannot be easily automated and will need manual chart review over the past 90 days by an expert.

### 1.11 SOFA with Richmond Agitation-Sedation Scale [SOFA _RASS_] Score

One of the difficulties associated with the SOFA score is that the nervous system component requires the calculation of GCS, which has been shown to be less reliably measured, resulting in a miscalculation or inability to calculate due to missing components in medical records. This has resulted in some modifications of the SOFA in which the neurological component is left out (mSOFA) [11], which can affect the efficacy of the scoring system. The Richmond Agitation-Sedation scale (RASS) is proposed to be reliable for arousal assessment in critically ill patients, including those on mechanical ventilation in whom GCS is difficult to measure. *Vasilevskis et al.* [33] showed that the SOFA_RASS_ (AUC of 0.814) was as good as the traditional SOFA score (0.799) in predicting ICU and in-hospital mortality in medical and surgical ICU patients.

### 1.12 Pediatric SOFA [pSOFA] Score

The SOFA score is mostly based on data in the adult population and is not adjusted for age, making it unsuitable for use in the pediatric population. The pediatric version of the SOFA (pSOFA) score was developed from the original SOFA score by using age-adjusted cut-offs in the cardiovascular and renal components along with extension of the respiratory criteria to include SpO2:FiO2 as an alternate non-invasive surrogate for lung injury. Studying Sepsis-3 definitions in critically ill children, *Matics et al.* reported that the maximum pSOFA score predicted in-hospital mortality with an AUC of 0.94, performing similar or better than other pediatric organ dysfunction scores [34].

## CONCLUSION

Several SOFA based models and derivatives have been described in the literature. The applicability of certain SOFA-based models is limited to the certain specific patient population (*e.g*. CLIF-SOFA in patients with liver failure and SOFA-HM in patients with hematologic malignancies). The mCV-SOFA and Mex-SOFA being the most recent, their use and applicability have yet to be determined. The qSOFA and LqSOFA were devised as simplified versions of the SOFA for easy use by most medical personnel in the emergency department setting. The pSOFA score employs almost the same variables with some age-adjusted cut-offs for the pediatric population.

## FUTURE DIRECTION

Rapid advances in clinical medicine and especially in critical care medicine have made the original SOFA score challenging to generalize and perhaps less pertinent. Incorporating the latest evidence-based medicine in order to update the components of the original SOFA score is the need of the hour and will help improve its prognostic value. However, the rapidity of development in critical care has to be tempered against the many measures that the ICU has adopted in the past that have subsequently proven futile. The de-adoption of the pulmonary artery catheter, decreased use of sedation, sedation vacations, *etc*. are illustrations of how these publications challenged the current practice at the time [35-37]. As we work towards modifications on the SOFA scores, we need to be aware of the fact that constant vigilance and periodic quality improvement on the current body of evidence will not only serve to upgrade the SOFA score but make it truly applicable across populations and continents. The gaps identified in this manuscript could be a focus on researching missing updates on SOFA components. Also, these updates were done at different time points at different institutions with a diverse patient cohort. This work has given us insights to put together most if not all updates in SOFA score and probably validate it in a large database a single institution and validate it in multisite database.

## Figures and Tables

**Table 1:** Type of modified sequential organ failure assessment score-based models described in literature.

**SOFA based Scoring Systems**	**Respiratory**	**Cardiovascular**	**Neurological**	**Hepatic**	**Renal**	**Coagulation**	
Original SOFA Score	- PaO2/FiO2 ratio - Respiratory support considered for values 3 and 4	- MAP<70 mm Hg - Use of vasopressors & dobutamine	- Glasgow coma scale	- Bilirubin (mg/dl or umol/L)	- Creatinine (mg/dl or umol/L) - Urinary output	- Platelets (x 1000/mm3)	
Modified SOFA (mSOFA)	- Respiratory support not included	- Blood pressure not included - Number of vasopressors	--	- Bilirubin (mg/dl)	- Creatinine (mg/dl)	Same as original SOFA	
Modified SOFA (MSOFA)	- SpO2/FiO2 ratio - Respiratory support considered for values 3 and 4	Same as original SOFA	Same as original SOFA	- Scleral icterus - Presence of jaundice	Same as original SOFA	--	
Modified cardiovascular SOFA (mCV-SOFA)	Same as original SOFA	- Lactate levels - Shock index - Number of vasopressors	Same as original SOFA	Same as original SOFA	Same as original SOFA	Same as original SOFA	
Extra-renal SOFA	Same as original SOFA	Same as original SOFA	Same as original SOA	Same as original SOFA	--	Same as original SOFA	
Chronic Liver Failure (CLIF-SOFA)	- PaO2/FiO2 ratio - SpO2/FiO2 ratio - Respiratory support not included	- Use of vasopressors	- Presence of grade II or IV hepatic encephalopathy	- Bilirubin (mg/dl)	- Creatinine (mg/dl) - Renal replacement therapy	- Platelets (x 1000/mm3) - International normalized ratio	
Mexican SOFA (Mex SOFA)	- SpO2/FiO2 ratio - Respiratory support considered for values 3 and 4	Same as original SOFA	--	Same as original SOFA	Same as original SOFA	Same as original SOFA	
Quick SOFA (qSOFA)	- Respiratory rate ≥22/min	- Systolic blood pressure ≤100 mmHg	- Change in mental status	--	--	--	
qSOFA and Plasma Lactic Acid Levels (LqSOFA)	- Respiratory rate ≥22/min	- Systolic blood pressure ≤100 mmHg - Lactate levels	- Change in mental status	--	--	--	
SOFA in Hematological Malignancies (SOFA-HM)	Same as original SOFA	Same as original SOFA	Same as original SOFA	Same as original SOFA	Same as original SOFA	Same as original SOFA	
	Additional System - Infection: Documented infection within 90 days						
SOFA with Richmond Agitation-Sedation scale (SOFA _RASS_)	Same as original SOFA	Same as original SOFA	Richmond Agitation-Sedation scale	Same as original SOFA	Same as original SOFA		Same as original SOFA
Pediatric SOFA (pSOFA)	- PaO2/FiO2 ratio OR SpO2/FiO2 ratio - Respiratory support considered for values 3 and 4	- MAP (age-adjusted) - Vasoactive agents (age-adjusted)	Same as original SOFA	Same as original SOFA	- Creatinine (age-adjusted)		Same as original SOFA

**Table 2:** Values of sequential organ failure assessment score described in literature.

**SOFA score values**	**Definition**
Total maximum SOFA	Sum of highest scores per individual organ system during entire ICU stay
Maximum SOFA	Highest total SOFA score measured in a pre-specified time interval
Mean SOFA	Average of all total SOFA scores in pre-specified time interval
Delta SOFA	Difference of total maximum and admission SOFA

SOFA: Sequential Organ Failure Assessment Score.

**Table 3:** Quick Sequential Organ Failure Assessment (qSOFA) score [21].

**qSOFA (Quick SOFA) Criteria**	**Points**
**Respiratory rate ≥22/min**	1
**Change in mental status**	1
**Systolic blood pressure ≤100 mmHg**	1

Table adapted from: Singer M, Deutschman CS, Seymour CW, Shankar-Hari M, Annane D, Bauer M, Bellomo R, Bernard GR, Chiche JD, Coopersmith CM *et al*: The Third International Consensus Definitions for Sepsis and Septic Shock (Sepsis-3). Jama 2016, 315(8):801-810.

## LIST OF ABBREVIATIONS

SOFA: = Sequential Organ Failure Assessment
mSOFA: = modified Sequential organ failure assessment
MSOFA: = Modified Sequential Organ Failure A
mCV SOFA: = modified Cardiovascular Sequential organ failure assessment
CLIF-SOFA: = Chronic Liver Failure Sequential Organ Failure Assessment
Mex-SOFA: = Mexican Sequential Organ Failure Assessment
qSOFA: = quick SOFA
LqSOFA: = Lactic acid quick SOFA
SOFA HM: = SOFA in Hematological Malignancies
SOFA _RASS_: = SOFA with Richmond Agitation-Sedation Scale
APACHE II: = Acute Physiologic and Chronic Health Evaluation II score
SAPS II: = Simplified Acute Physiology II score
RRT: = Renal Replacement Therapy
INR: = International Normalized Ratio
PaO_2_: = Partial Pressure Arterial Oxygen
FiO_2_: = Fraction of Inspired Oxygen
SpO_2_: = Oxygen Saturation
