# Supplementary material for: Current Utility of Sequential Organ Failure Assessment Score: A Literature Review and Future Directions
Source: Open Respir Med J. 2021 Apr 13;15:1–6. doi: 10.2174/1874306402115010001 (PMC8227444; doi:10.2174/1874306402115010001)
Supplement: Supplementary file 1 — Supplementary material is available on the publishers web site along with the published article. [file TORMJ-15-1_Sp.pdf]

1874-3064/21 *Send Orders for Reprints to reprints@benthamscience.net*

The Open Respiratory Medicine Journal

Content list available at: https://openrespiratorymedicinejournal.com

Supplementary Material

Current Utility of Sequential Organ Failure Assessment Score: A Literature Review and Future Directions

Rahul Kashyap[1], Khalid M. Sherani[2,3], Taru Dutt[4], Karthik Gnanapandithan[5], Malvika Sagar[6], Saraschandra Vallabhajosyula[7], Abhay P. Vakil[6,7] and Salim Surani[3,8,*]

[1]*Department of Anesthesiology and Perioperative Medicine, Mayo Clinic, Rochester, MN 55905, USA*
[2]*Department of Internal Medicine, Jamaica Hospital Medical Center, Jamaica, NY 11418, USA*
[3]*Corpus Christi Medical Center, Corpus Christi, TX 78411, USA*
[4]*Department of Neurology, Mayo Clinic College of Medicine, Mayo Clinic, Rochester MN, USA and Hennepin County Medical Center, Minneapolis, MN 55905, USA*
[5]*Department of Internal Medicine, Yale-New Haven Hospital and Yale University School of Medicine, New Haven, CT 06510, USA*
[6]*Department of Pediatrics, McLane Children's Hospital, Baylor Scott and White Health, Temple, TX 76502, USA*
[7]*Critical Care Medicine, Mayo Clinic College of Medicine, Mayo Clinic, Rochester, MN 55905, USA*
[8]*Texas A&M University System Health Science Center, Bryan, TX 77807, USA*

Article History	Received: October 2, 2020	Revised: December 13, 2020	Accepted: January 13, 2021

Table S1. Sequential Organ Failure Assessment score from original article [8].

-	SOFA Score				
-	**0**	**1**	**2**	**3**	**4**
Respiration	-	-	-	-	-
$PaO_2/Fi0_2$ (torr)	>400	≤400	≤300	≤200	≤100
	-	-	-	With respiratory support	With respiratory support
Coagulation	-	-	-	-	-
Platelets ($x10^3/mm^2$)	>150	≤150	≤100	≤50	≤20
Liver	-	-	-	-	-
Bilirubin (mg/dL)	<1.2	1.2-1.9	2.0-5.9	6.0-11.9	>12.0
(µmol/L)	<20	20-32	33-101	102-204	>204
Cardiovascular	-	-	-	-	-
Hypotension	No	MAP <	Dopamine ≤6	Dopamine > 6	Dopamine > 15
	hypotension	70mm Hg	or dobutamine	or epi ≤ 0.1	or epi > 0.1
	-	-	(any dose)*	or norepi ≤ 0.1*	or norepi > 0.1*
Central Nervous System	-	-	-	-	-
Glasgow Coma Score	15	13-14	10-12	6-9	<6
Renal	-	-	-	-	-
Creatinine (mg/dL)	<1.2	1.2-1.9	2.0-3.4	3.5-4.9	>5.0
(µmol/L)	<110	110-170	171-299	300-440	>440
or urine output	-	-	-	or <500 mL/day	or <200 mL/day

epi, epinerphine; norepi, norepinephrine.
*Adrenergic agents administered for at least 1 hr (doses are given in µg/kg/min)
To convert torr to kPa, multiply the value by 0.1333.

Table adapted from: Vincent JL, Moreno R, Takala J, Willatts S, De Mendonca A, Bruining H, Reinhart CK, Suter PM, Thijs LG: The SOFA (Sepsis-related Organ Failure Assessment) score to describe organ dysfunction/failure. On behalf of the Working Group on Sepsis-Related Problems of the European Society of Intensive Care Medicine. Intensive Care Med 1996, 22(7):707-710.

© 2021 Kashyap *et al.*

This is an open access article distributed under the terms of the Creative Commons Attribution 4.0 International Public License (CC-BY 4.0), a copy of which is available at: https://creativecommons.org/licenses/by/4.0/legalcode. This license permits unrestricted use, distribution, and reproduction in any medium, provided the original author and source are credited.
